# Loss of PRDX6 Aborts Proliferative and Migratory Signaling in Hepatocarcinoma Cell Lines

**DOI:** 10.3390/antiox12061153

**Published:** 2023-05-25

**Authors:** Daniel J. Lagal, María J. López-Grueso, José R. Pedrajas, Thomas L. Leto, J. Antonio Bárcena, Raquel Requejo-Aguilar, C. Alicia Padilla

**Affiliations:** 1 Department Biochemistry and Molecular Biology, University of Córdoba, 14071 Córdoba, Spain; b32larud@uco.es (D.J.L.); q02logrm@uco.es (M.J.L.-G.); bb1barua@uco.es (J.A.B.); 2 Group of Biochemistry and Cell Signaling in Nitric Oxide, Department of Experimental Biology, Institute of Research in Olive Groves and Olive Oils, University of Jaén, 23071 Jaén, Spain; pedrajas@ujaen.es; 3 Laboratory of Clinical Immunology and Microbiology, National Institute of Allergy and Infectious Diseases, National Institutes of Health, Rockville, MD 20892, USA; tleto@niaid.nih.gov; 4 Maimónides Biomedical Research Institute of Córdoba (IMIBIC), 14004 Córdoba, Spain

**Keywords:** redoxins, lipid peroxidation, mitochondrial dysfunction, GSK3β, epithelial–mesenchymal transition (EMT)

## Abstract

Peroxiredoxin 6 (PRDX6), the only mammalian 1-Cys member of the peroxiredoxin family, has peroxidase, phospholipase A2 (PLA2), and lysophosphatidylcholine (LPC) acyltransferase (LPCAT) activities. It has been associated with tumor progression and cancer metastasis, but the mechanisms involved are not clear. We constructed an SNU475 hepatocarcinoma cell line knockout for PRDX6 to study the processes of migration and invasiveness in these mesenchymal cells. They showed lipid peroxidation but inhibition of the NRF2 transcriptional regulator, mitochondrial dysfunction, metabolic reprogramming, an altered cytoskeleton, down-regulation of PCNA, and a diminished growth rate. LPC regulatory action was inhibited, indicating that loss of both the peroxidase and PLA2 activities of PRDX6 are involved. Upstream regulators MYC, ATF4, HNF4A, and HNF4G were activated. Despite AKT activation and GSK3β inhibition, the prosurvival pathway and the SNAI1-induced EMT program were aborted in the absence of PRDX6, as indicated by diminished migration and invasiveness, down-regulation of bottom-line markers of the EMT program, MMP2, cytoskeletal proteins, and triggering of the “cadherin switch”. These changes point to a role for PRDX6 in tumor development and metastasis, so it can be considered a candidate for antitumoral therapies.

## 1. Introduction

Reactive oxygen species (ROS) are molecular messengers in normal metabolism and cell signaling [1] that can be generated in mitochondrial oxidative metabolism, endoplasmic reticulum during protein folding, or as products of the normal activity of membrane-bound NADPH-dependent oxidases (NOX) [2,3]. Tumors have high levels of ROS that can mediate cellular proliferation, evasion of apoptosis, tissue invasion, and angiogenesis [4,5]. To promote these pro-metastatic functions, cancer cells depend on antioxidant systems to tolerate excessive ROS levels. Consequently, some anti-cancer therapies are based on antioxidant inhibition [6].

PRDX6 is unique within the peroxiredoxin family, as it is capable of reducing phospholipid hydroperoxides [7], whose product, a hydroxy-fatty acid, plays other roles in cell signaling. It also has both Ca^2+^-independent phospholipase A2 (aiPLA2) and lysophosphatidylcholine acyltransferase (LPCAT) activities [8]. The products of these activities can also act as lipokines [9]. Paradoxically, PRDX6 also has “pro-oxidant” activity, as it supports the activities of some NADPH oxidase enzymes that release ROS outside the cell [10,11].

PRDX6 has been considered a tumor promoter [12,13,14,15]. Interestingly, PRDX6 overexpression was correlated with a more invasive phenotype in colorectal adenoma [16], esophageal squamous cell carcinoma [17], and breast cancer [18]. PRDX6 has been implicated in cell migration through interaction with NOX1 in HCT-116 cancer cells, specifically through aiPLA2 activity [11]. The same behavior of invasiveness has been identified in lung cancer cells due to aiPLA2 activity, whereas peroxidase activity has been implicated in the growth of lung cancer cells [19], and its overexpression promoted proliferation, invasion, and migration of A549 cells in vitro and in vivo [20].

Epithelial–mesenchymal transition (EMT) is a reversible phenotypic conversion in which cells lose adherence junctions and polarity and gain motility and invasion capacity [21]. Some evidence has linked PRDX6 to EMT, but additional research on its mechanism of metastatic action is needed to identify novel therapeutic targets and strategies against cancer. We previously observed that down-regulation or deletion of PRDX6 by CRISPR/Cas9 technology slowed proliferation in HepG2 cells, affected metabolism, and promoted cell cycle arrest by affecting the oxidation state of the cell cycle protein PCNA [22,23]. However, EMT could not be studied in these cells because their epithelial nature prevents them from migrating (unpublished observations). Hence, to explore the role of PRDX6 in cell migration and invasiveness, we studied the effect of eliminating PRDX6 in the mesenchymal cell line SNU475. We show for the first time that, in addition to the antiproliferative effects observed in HepG2 cells, depletion of PRDX6 also reduces the migration and invasiveness capacities of SNU475 cells in vitro, which involves AKT-dependent inactivation of GSK3β.

## 2. Materials and Methods

### 2.1. Materials and Reagents

All reagents were of analytical grade and were purchased from Sigma (St. Louis, MO, USA) unless otherwise specified. Cell culture dishes and flasks were from TPP (Switzerland). ECL was from GE Healthcare (Wauwatosa, WI, USA). Antibodies against PRDX6 were from Mybiosource (Cambridge, UK). Antibodies against N-cadherin, caspase-3, E-cadherin, PCNA, GSK3β, pGSK3β^S9^, and pAKT^S473^ were purchased from Cell Signaling (Danvers, MA, USA). Antibodies against AKT and actin were from Santa Cruz (Dallas, TX, USA). HA-flag antibody was from Thermo Fisher Scientific (Waltham, MA, USA), and complex I antibody and secondary antibodies conjugated with horseradish peroxidase were from Sigma. Secondary antibodies conjugated with Alexa Fluor 488 and 647 fluorophores and Alexa Fluor 488 phalloidin were from Abcam (Cambridge, UK), and Matrigel^®^ Matrix basement membrane was from Corning (New York, NY, USA).

### 2.2. Cell Lines

HepG2 (HB-8065™) and SNU475 (CRL-2236) standard cell lines were obtained from American Type Culture Collection (ATCC; LGC Standards, S.L.U., Barcelona, Spain). They were grown in aseptic conditions at pH 7.4 in an incubator with a 5% CO_2_ atmosphere and 37 °C using EMEM and RPMI 1640 media (Sigma). Both were supplemented with 2.2 g/L NaHCO_3_, 1 mM sodium pyruvate, 10% fetal bovine serum, 100 U/L penicillin, 100 μg/mL streptomycin, and 0.25 μg/mL amphotericin. For preparation of the cell-free extracts, cells were detached with trypsin-EDTA or by scraping, depending on the analysis.

### 2.3. Creation of a Peroxiredoxin 6-Deficient SNU475 Hepatocarcinoma Cell Line (SNU475^PRDX6−/−^) Using the CRISPR/Cas9 Methodology

An SNU475 knockout for the PRDX6 cell line was generated using the CRISPR/Cas9 technique with the same protocol and specific gRNA described for the HepG2 cell line [23], modified with an increased transfection time up to 72 h to improve efficiency. The individual clones were grown and analyzed for the presence of PRDX6 using Western blot with PRDX6-specific antibodies. The presence of PRDX6 was also evaluated using immunofluorescence with the same antibody with a 1:200 dilution and the Alexa anti-Ig rabbit 488 antibody with a 1:100 dilution. Additionally, Sanger sequencing was performed with specific primers to the exon 3 region of the *prdx6* gene at the SCAI (University of Córdoba).

### 2.4. Plasmid Transfection

The pcDNA3.1 plasmid containing PRDX6 cDNA or GFP cDNA was used to transfect SNU475^PRDX6+/+^ and SNU475^PRDX6−/−^ cells. A total of 2 × 10^5^ cells were seeded in 6-well plates and were transfected for 5 h upon reaching 80% of confluence using the lipofectamine 3000 kit, according to the manufacturer’s guidelines (Thermo Fisher Scientist), with 2.4 µg of plasmid DNA and a ratio of 1:2:2 (DNA (µg):P3000 reagent (µL):lipofectamine (µL)). 

### 2.5. Measurement of Protein, ROS, and Lipid Peroxidation 

Protein concentration was determined using a BCA Assay Kit (Thermo Fisher Scientist) with BSA as the standard. ROS were measured using the DCFDA/H2DCFDA method and a commercial ROS Assay Kit (Canvax Biotech S.L., Córdoba, Spain). Lipid peroxidation was quantified using a TBARS Assay Kit (Canvax Biotech S.L.), based on the reaction of malondialdehyde (MDA) derived from peroxidized lipids with thiobarbituric acid (TBA).

### 2.6. SDS-PAGE and Western Blot

SDS-PAGE was performed in 10–15% acrylamide gels, and electrophoresis was carried out in a Mini-PROTEAN Tetra Cell system (BioRad, Hercules, CA, USA) for 1 h 30 min at 120 V. After electrophoresis, the proteins were transferred to a nitrocellulose membrane using a wet transfer system for 1 h 45 min at 300 mA. Transfer and protein load were checked by staining with Ponceau reagent. Membranes were incubated overnight at 4 °C with the dilutions of primary antibodies, as recommended by the manufacturer. They were then washed with TBS-T and incubated with the corresponding peroxidase-conjugated secondary antibody (anti-rabbit, anti-goat, or anti-mouse) used at 1:8000 dilution. The chemiluminescent signal produced by the ECL reagent (GE Healthcare, Chicago, IL, USA) was detected in a ChemiDoc image analyzer (Bio-Rad) and quantified with ImageJ software using actin as a reference for protein normalization.

### 2.7. Immunofluorescence

SNU475 cells (3.5 × 10^3^) were seeded on cover glass for confocal microscopy with regular medium and allowed to grow overnight. Then, the cells were fixed with 4% PFA in PBS solution for 20 min at room temperature and washed with PBS for 10 min twice. Permeabilization was performed with 0.3% Triton X-100 in PBS solution for 10 min at room temperature. Blocking was carried out with 2% BSA in PBS solution for 1 h. Primary antibody was incubated overnight at 4 °C in a 2% BSA solution. Then, the cells were washed with PBS for 5 min three times. Fluorescence secondary antibody (1:200) or phalloidin marker (1:1000) were incubated for 1 h in a 2% BSA solution, followed by washes as in the previous step. Finally, the cells were mounted with mounting medium with DAPI (Abcam). The cells were visualized using a confocal microscope (LSM 710) at the IMIBIC microscopy service (Córdoba, Spain) or a conventional fluorescence microscope (Leica DM6B) (Wetzlar, Germany).

### 2.8. Extracellular Flux Analysis of Mitochondrial Respiration and Glycolytic Function

Mitochondrial respiration using an Agilent Seahorse XF24 Analyzer and an Agilent Seahorse XF Cell Mito Stress Test (Agilent Seahorse Bioscience, Santa Clara, CA, USA) was assayed as previously described [23]. SNU475 cells were seeded at 2.5 × 10^4^ cells per well in a Seahorse 24-well XF Cell Culture microplate. The mitochondrial function of the cells was analyzed using sequential injections of the modulators oligomycin (1 µM), carbonyl cyanide-p-trifluoromethoxyphenylhydrazone (FCCP, 0.25 µM), and rotenone/A (0.5 µM). Measurement of oxygen consumption rate (OCR) was performed at the beginning and after the addition of each modulator. Basal respiration is the OCR at the beginning of the experiment in the absence of modulators and reflects the energetic demand of the cells under basal conditions, which includes proton leak not coupled to ATP production; OCR after the addition of oligomycin is the basal respiration not coupled to ATP production, and the difference between basal respiration and proton leak is a measure of the actual oxygen consumption coupled to ATP production; and maximal respiration is attained after the addition of the uncoupler FCCP when the respiratory chain operates at maximum capacity. The difference between maximal and basal respiration reflects the spare respiratory capacity or energetic reserve of the cells, and non-mitochondrial respiration is the OCR that persists after the addition of rotenone and antimycin A. Data were normalized to protein concentration determined at the end of the assay.

An Agilent Seahorse XF Cell Glycolysis Stress Test was applied to SNU475^PRDX6+/+^ and SNU475^PRDX6−/−^ cells. The protocol was designed as Cell Mito Stress, but the XF assay medium was supplemented with 2 mM sodium pyruvate and 2 mM glutamine, but no glucose. The glycolytic function of the cells was analyzed using sequential injections of glucose (10 mM), oligomycin (1 mM), and 2-deoxy-glucose (50 mM). Measurement of the extracellular acidification rate (ECAR) was performed at the beginning and after the addition of each modulator. The glycolysis, glycolytic capacity, glycolytic reserve, and non-glycolytic acidification parameters were determined following the manufacturer’s guidelines. Data were also normalized to protein concentration at the end of the assay.

### 2.9. Determination of the Enzymatic Activities of Mitochondrial Complexes 

The activity of the I/III complexes was measured in 20 mM KPi buffer and 0.1 mM EDTA, pH 8.0, supplemented with 2 mM KCN. After equilibration for 10 min, cytochrome c (50 μM) was added, and its reduction was determined at 550 nm. After that, the remaining activity of complex I was assayed at 550 nm after adding 10 μM of rotenone [24].

### 2.10. Cell Proliferation, Viability, and Cell Cycle Analysis

SNU475^PRDX6+/+^ and SNU475^PRDX6−/−^ cells (3.5 × 10^4^) were seeded in 24-well plates and allowed to grow to generate growth curves of counted cells at 24, 48, and 72 h. For cell cycle analysis, 5 × 10^5^ of these cells were seeded in a 60 cm^2^ plate and incubated for 48 h. After that, a 3 h pulse with BrdU (10 mg/mL) was carried out for BrdU incorporation, as determined using the APC BrdU Flow Kit (Becton Dickinson Biosciences, Franklin Lakes, NJ, USA). The proportions of cell cycle phases were determined using flow cytometric analyses of 7-AAD-stained SNU475 cells. The cytometer used was the BD Accuri^TM^ C6 Plus (BD Biosciences). The data were processed with BD Accuri™ C6 Plus 1.0.34 software (BD Biosciences).

### 2.11. Cell Death Assay

The early and late stages of apoptosis and necrosis were determined using an Annexin V Apoptosis Detection Kit through flow cytometry (Canvax Biotech S.L.). SNU475^PRDX6+/+^ and SNU475^PRDX6−/−^ cells (2 × 10^5^) were seeded in 6-well plates and allowed to grow for 48 h. Then, the cells were detached and washed with PBS once. The cells were marked with labeled Annexin V, which was detected using a FITC laser. Propidium iodide was also added and detected with PE laser following the manufacturer’s guidelines. Control of cell death was carried out by heating SNU475 cells at 65 °C for 15 min. The cytometer used was the BD Accuri^TM^ C6 Plus. The data were processed with integrated BD Accuri™ C6 Plus Software (BD Biosciences).

### 2.12. Metalloproteinase Activity Detection Assay

SNU475^PRDX6+/+^ and SNU475^PRDX6−/−^ cells (2 × 10^5^) and HepG2^PRDX6+/+^ and HepG2^PRDX6−/−^ cells (4 × 10^5^) were seeded in 6-well plates and allowed to grow for 48 h in regular medium. Then, the cells were washed with PBS solution twice, and free serum medium was added. The cells were incubated in this medium for at least 48 h. Then, the medium was collected and kept at −80 °C until the day of the assay, and the cells were lysed and collected by scraping. Samples were charged on gelatin discontinuous gels as previously described [25], and electrophoresis was carried out for 2 h at 120 V with normal electrophoresis running buffer. After electrophoresis, the gels were incubated in a 25% Triton X-100 solution for 30 min with gentle shaking, and metalloproteinase activity was developed for 30 min at room temperature, followed by 30 h at 37 °C. Once incubation was concluded, the gels were washed twice with water for 5 min with shaking. The proteinase-digested clear bands were detected by staining gels with a Coomassie blue solution for 1 h at room temperature, followed by a long incubation in destaining solution. The integrative intensity of the clear bands was measured using ImageJ.

### 2.13. Wound Healing Assay

A wound healing assay was used to determine cell migration. To do this, 6.5 × 10^4^ cells per well were seeded into 2-well Ibidi silicone culture inserts (Ibidi LLC, Fitchburg, WI, USA), which provided a defined cell-free gap. When the cells reached a confluence of 90%, the silicone insert was removed, creating a 500 µm cell-free gap. Unattached cells were washed with serum-free medium twice, and regular medium was added to allow cell migration. Cell migration progressed until 90% of the wound gap was closed (24 h). Then, the cells were fixed with 100% methanol for 20 min at room temperature and stained with 0.5% crystal violet dissolved in 25% methanol. Wound closure was measured by quantifying the free-cell area with ImageJ software, and the gap closure index (GCI) was calculated using this formula:

(1) $$GCl=1-\frac{GT}{G0}$$

where GT is the gap size computed at the end point time T, and G0 is the initial starting point gap size at T = 0. A GCI of 0, 0.5, or 1 indicates that the gap has not changed and is identical in area to the initial gap, has closed by 50%, or has fully closed at time T, respectively.

### 2.14. Transwell Invasion Assay 

A transwell assay was applied to determine cell invasion capacity. BioCoat^®^ Matrigel^®^ Matrix 24-well plates with 8 μm pore size (Corning) were allowed to reach room temperature. The transwell chambers were hydrated with 500 mL of serum-free culture medium in both the upper and lower transwell chambers and incubated for 2 h at 37 °C and 5% CO_2_. Then, the medium was removed, regular medium was added to the lower chamber, and 1 × 10^5^ SNU475^PRDX6+/+^ or SNU475^PRDX6−/−^ cells were seeded with regular medium on the upper chamber. The cells were able to invade for 24 h. After that time, the upper chamber medium and non-invasive cells were removed with a cotton swab. Then, translocated cells were fixed with cold methanol for 30 min and stained with 0.5% crystal violet dissolved in 25% methanol. The invasive cells were then analyzed with a Leica optical microscope (Wetzlar, Germany).

### 2.15. Global Proteome Analysis 

The experiments were routinely carried out at 20,000 cells/cm^2^. Cell extracts were obtained by lysis with sea sand in Tris-HCl (50 mM), pH 8.5, containing 2 mM DTT. The cells were vortexed and incubated on ice for 5 min. Then, the extracts were centrifuged at 400× *g*, and the supernatants were collected and centrifuged at 8000× *g* and stored at −20 °C until use. Four biological replicas of each sample were analyzed. Proteomics analyses were performed at the Proteomics Facility (SCAI) at the University of Córdoba. The mass spectrometer used was the Thermo Orbitrap Fusion (Q-OT-qIT, Thermo Scientific) with a 2 h gradient in a nano-UHLC Ultimate 3000 (Dionex-Thermo Scientific) chromatograph. The setting of the instrument was as previously described [26]. MaxQuant (v1.5.7.0) and Perseus (v1.5.6.0) software were used to analyze the different MSe runs in quadruple. Proteins were identified by searching raw data against the human UniprotKB/Swiss-Prot protein database (February 2018 version). Functional enrichment analysis was carried out using the Ingenuity Pathways Analysis tool (IPA-Ingenuity Systems, www.ingenuity.com, access date 17 November 2022) and DAVID enrichment analysis tools [27].

### 2.16. Statistics

Where appropriate, results are expressed as mean ± SD of at least three independent experiments. Statistical analysis of the data was performed using the Mann-Whitney test, unpaired Student’s *t*-test, or two-way ANOVA, depending on the experiment design, as detailed in the figure legends, with the threshold for statistically significant differences set at a *p* adjusted < 0.05 value.

## 3. Results

### 3.1. Creation and Characterization of SNU475 Hepatocarcinoma Cell Line Lacking PRDX6 

Cells were transfected following the CRISPR/Cas9 technique as described in the Materials and Methods section, and the efficiency was calculated, resulting in a value of 6.7% (Supplementary Figure S1A). After transfection, limiting dilutions were carried out in 96-well plates, and more than 100 clones were isolated. Analysis by immunofluorescence and Western blot with specific antibodies against PRDX6 confirmed the absence of PRDX6 in one of these clones (Supplementary Figure S1B). This clone was established as a PRDX6 knockout SNU475 cell line for further experiments. 

Sanger sequencing of genomic DNA from these SNU475^PRDX6−/−^ cells (KO) with specific primers showed a 17 nucleotide deletion in one allele and insertion of one cytosine in the other (Supplementary Figure S1C,D). Thus, the *prdx6* gene disruption was biallelic and heterozygous, in which the two alleles were repaired differently.

### 3.2. Differential Quantitative Proteomic Analysis of SNU475^PRDX6+/+^ and SNU475^PRDX6−/−^ Cells

The differential proteome of WT and KO SNU475 cells (Supplementary Table S1A) was systems analyzed using the IPA and DAVID bioinformatics tools. According to DAVID, tRNA-aminoacylation, protein biosynthesis, and nucleo-cytoplasmic transport were up-regulated in the SNU475-KO cells, while lipid metabolism, specifically the cholesterol pathway, including the mevalonate branch and terpenoid backbone biosynthesis, the cytoskeleton, and “the role of GTSE1 in G2/M progression after G2 checkpoint” were down-regulated (Table 1A).

According to IPA, the most significantly affected (*p*-value < 10^−9^) upstream regulators were TP53 and NRF2, which were slightly inhibited, and MYC, ATF4, HNF4A, and HNF4G, which were highly activated (Table 1B). Six proteasome proteins were significantly down-regulated (Supplementary Table S1B): PSMB8, PSMB10, PSME1, and PSME2 are involved in immunoproteasome assembly and antigen processing, while PSMA1 and PSM3 are components of the 20S complex; the former is overexpressed in liver tumors, and the latter mediates ubiquitin-independent protein degradation. These changes are part of the BAG2 signaling pathway activation, according to IPA (Table 1C).

The implications of these proteomic changes in particular proteins will be discussed in the appropriate context in the following sections.

### 3.3. Oxidative Stress Levels Increased in Hepatocarcinoma Cell Lines Lacking PRDX6 

**Figure 1:**
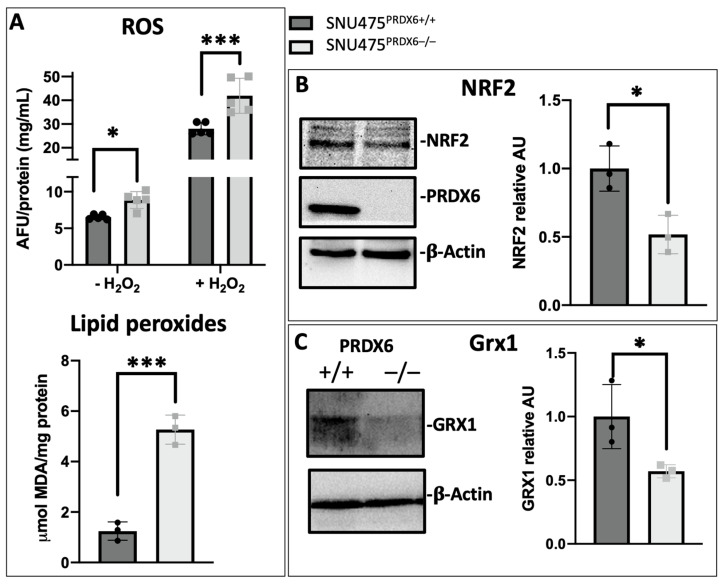
Oxidative stress induced by PRDX6 deficiency in SNU475 cells. (**A**) Top: ROS levels in basal and 100 μM H_2_O_2_-treated cells expressed as arbitrary fluorescence units (AFU) in the DCFDA assay normalized for protein concentration (n = 5); bottom: peroxidized lipid levels were determined by TBARS assay measuring the production of malondialdehyde (MDA) (n = 3). (**B**) NRF2 levels determined by Western blot; a representative blot is shown, and quantitative results are expressed as arbitrary units (AU) normalized to β-actin and relative to SNU475^PRDX6+/+^ levels (n = 3). (**C**) Normalized and relative to WT Grx1 levels determined using Western blot, expressed as arbitrary units (AU). (Black circles and grey squares are the individual values of the different replicas of WT and KO cells, respectively; Student’s *t*-test,* *p*-value ≤ 0.05; *** *p*-value ≤ 0.001).

ROS levels and peroxidized lipids were significantly higher in SNU475^PRDX6−/−^ cells than in SNU475^PRDX6+/+^ cells (Figure 1A), indicating that antioxidant defenses were down-regulated in cells lacking PRDX6. Actually, we detected lower levels of the antioxidant master regulator NRF2 (Figure 1B) and of several proteins under its control, including thioredoxin reductase and glutathione transferases mu1 and 3, but higher levels of the stress proteins squestosome 1 and stress-induced phosphoprotein 1 (Supplementary Table S1B), which is indicative of NRF2 deficiency (Table 1C).

Additionally, an important antioxidant enzyme involved in redox regulation, independent of NRF2, glutaredoxin1 (Grx1), which has deglutathionylating activity, also decreased significantly (Figure 1C). Similar results were observed in HepG2 cells [23].

Lipid peroxidation is consistent with the activation of the ATF4 transcriptional regulator observed in proteomic analyses (Table 1C), and these data all together should be a consequence of PRDX6 deficiency and an impaired ability to reduce phospholipid hydroperoxides.

### 3.4. PRDX6 Deficiency Led to Mitochondrial Dysfunction and Metabolic Alterations in SNU475 Cells

**Figure 2:**
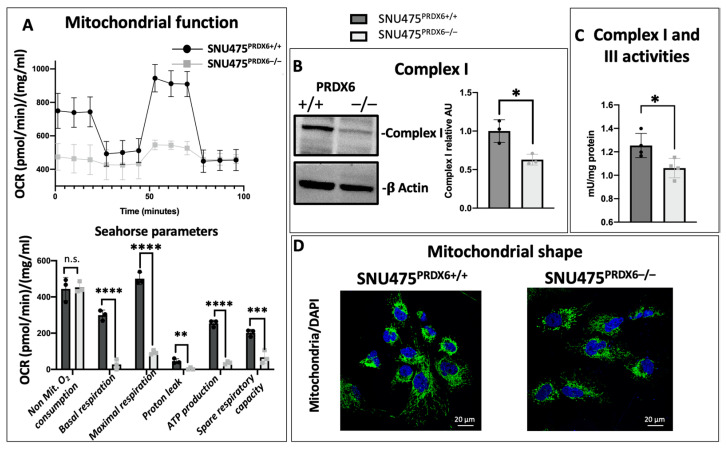
Mitochondrial dysfunction caused by PRDX6 deletion. (**A**) Representative recording of oxygen consumption rate (OCR) during extracellular flow analysis for the Mito Stress Test and histogram plots of the calculated non-mitochondrial O_2_ consumption, basal and maximum respiratory rate, H^+^ proton leak, ATP production rate, and respiratory capacity reserve. Both were normalized for protein content (n = 3). (**B**) Analysis of complex I (75 kDa) protein by Western blot, relative to WT and normalized by β-actin levels expressed as arbitrary units (AU) (n = 3). (**C**) Complex I & III enzymatic activity (n = 4), normalized for protein content. (**D**) Mitochondria visualization by immunofluorescence microscopy (nuclei have been stained with DAPI and appear blue; 488 mitochondrial marker antibody was stained green). (Multiple *t*-test, * *p*-value ≤ 0.05; ** *p*-value ≤ 0.005; *** *p*-value ≤ 0.001; **** *p*-value ≤ 0.0001, n.s. not significant).

Mitochondrial respiratory functionality was determined using in vivo extracellular flow analysis by Seahorse. All parameters, including basal and maximal respiration, respiratory reserve capacity, as well as ATP production and proton leakage, decreased by more than 50% in SNU475 cells lacking PRDX6 (Figure 2A). These alterations in mitochondrial respiration are consistent with the decreased mitochondrial complex I observed with Western blot and enzymatic activity (Figure 2B,C) and the spheroid-shaped appearance of mitochondria instead of the normal reticulated morphology, as observed with fluorescence microscopy (Figure 2D).

In addition, comparative IPA analysis showed activation of the transcription factor ATF4 in cells lacking PRDX6 (Table 1C). ATF4 regulates metabolic and redox processes under normal cellular conditions and is the master transcription factor during the integrated stress response (ISR). It is activated by increases in lipid hydroperoxides, which is likely to occur in dysfunctional mitochondria [28], which is what we observed in SNU475^PRDX6−/−^ cells. These results suggest that PRDX6 is regulating mitochondrial oxidative metabolism. 

**Figure 3:**
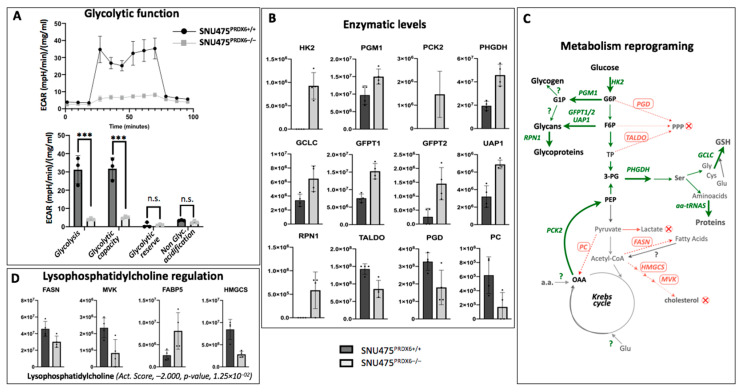
Metabolic reprogramming caused by PRDX6 deletion. (**A**) Representative recording of extracellular acidification rate (ECAR) during extracellular flow analysis following the protocol XF Glycolysis Stress Test for glycolytic function and histogram plots of the calculated glycolysis, glycolytic capacity, glycolytic reserve, and non-glycolytic acidification rates, normalized for protein content (n = 3). (**B**) Levels of 12 enzymes differentially expressed as quantified in the proteomic analysis are shown; intensity of the MS signal in area units for each protein, as calculated from areas of peptide peaks by the MaxQ algorithm, is shown in the Y axis (n = 4); data are presented in Supplementary Table S1B (Excel spreadsheet). (**C**) Scheme of interpreted metabolic flux changes according to enzymes up-regulated (names in green color, thick arrows) or down-regulated (names in red color, dotted arrows) in SNU475-KO cells compared to WT. (**D**) Levels of four enzymes of lipid metabolism known to be targets of lysophosphatidylcholine; the activation score calculated by IPA is shown underneath. (Multiple *t*-test, *** *p*-value ≤ 0.001, n.s. not significant).

Regarding glucose metabolism, knockout of PRDX6 strongly reduced the glycolytic capacity of cells (Figure 3A). The proteomic study found nine key glucose metabolism enzymes that were highly up-regulated in SNU475^PRDX6−/−^ cells: hexokinase 2 (HK2), phosphoglucomutase 1 (PGM1), phosphoenolpyruvate carboxykinase 2 (PCK2), phosphoglycerate dehydrogenase (PHGDH), glutamate–cysteine ligase catalytic subunit (GCLC), glutamine-fructose-6-phosphate transaminase 1/2 (GFPT1/2), UDP-N-acetylglucosamine pyrophosphorylase (UAP1), and dolicoyl oligosacaryl transferase (RPN1). Conversely, transaldolase (TALDO), phosphogluconate dehydrogenase (PGD), and pyruvate carboxylase (PC) were down-regulated (Figure 3B). PGM1 has recently been described as a suppressor of hepatocarcinoma progression by regulating glucose trafficking [29]. These enzymatic changes reveal pentose phosphate pathway (PPP) inhibition, glucose channeling from fructose-6-phosphate toward glycans, glycoprotein synthesis, and gluconeogenic metabolic flux from oxaloacetate, with diversion toward serine biosynthesis and glutathione synthesis and amino acid biosynthesis pathways therefrom (Figure 3C). These altered metabolic fluxes would explain the decrease in glycolytic flow and glucose-dependent respiratory capacity observed in Seahorse.

Lipid metabolism showed no changes in β-oxidation enzymes, but fatty acid synthase (FASN) and cholesterol biosynthesis were down-regulated in response to PRDX6 deprivation (Figure 3D). IPA analysis indicated that there was inhibition of the regulatory role of lysophosphatidylcholine (LPC) in SNU475^PRDX6−/−^ cells based on the changes observed in the four proteins (Figure 3D) involved in cholesterol biosynthesis, presumably through sterol regulatory element-binding protein 2 (SREBP2) [30].

Analysis of proteomic data on nitrogen metabolism showed no changes in nucleotide metabolism and marked activation of protein synthesis in SNU475^PRDX6−/−^ cells, as indicated by significant increases in several aminoacyl-tRNA synthetases (Table 1A,B).

### 3.5. PRDX6 Deficiency Decreased Proliferation, Altered the Cytoskeleton, Promoted Cell Cycle Arrest at G2/M Phase, and Affected the AKT/GSK3β Signaling Pathway

**Figure 4:**
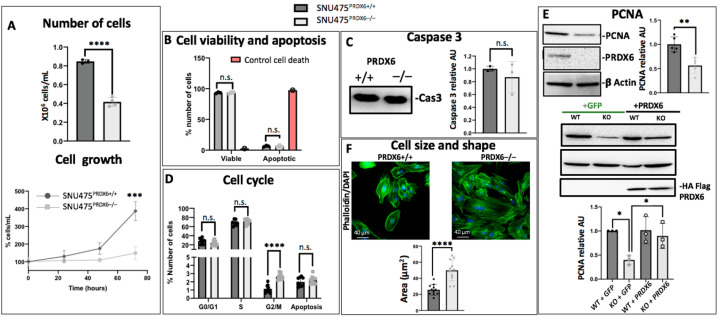
Growth, size, shape, and cell cycle of SNU475^PRDX6−/−^ cells. (**A**) Top: cell count at time 0 and after 1 week of growth (n = 6); bottom: proliferation rate after 24, 48, and 72 h of cell culture (n = 4). (**B**) Determination of cell death and cell viability by FITC-marked annexin V and propidium iodide detection (n = 3). (**C**) Levels of caspase 3 relative to WT determined by Western blot (n = 3). (**D**) Flow cytometry analysis showing the number of cells in the different cell cycle phases in WT and SNU475 KO cells (n = 9). (**E**) Top: analysis of PCNA protein using Western blot normalized by β-actin and relative to WT (n = 5); bottom: PCNA levels in SNU475^PRDX6−/−^ cells transfected with *pcDNA3.1 + prdx6* or *pcDNA3.1 + GFP*, determined using Western blot (n = 3). (**F**) Cell shape and cell size determination expressed by cellular area in μm^2^ using immunofluorescence of the cytoskeleton with 488-marked phalloidin (n = 13). Cell size was also checked by measuring cell area (µm^2^) in transmission electron micrographs. AU = Arbitrary units. Multiple *t*-test, * *p*-value ≤ 0.05; ** *p*-value ≤ 0.005; *** *p*-value ≤ 0.001; **** *p*-value ≤ 0.0001; n.s. not significant.

Cell proliferation was significantly lower in SNU475^PRDX6−/−^ cells compared with SNU475^PRDX6+/+^ cells (Figure 4A). This decreased proliferation was not attributed to increased cell death, since the viability did not decrease (Figure 4B) and there were no signs of apoptosis (Figure 4B–D).

PRDX6 deficiency in SNU475 cells caused alterations in the cell cycle, with arrest at the G2/M phase (Figure 4D), although to a lesser extent than HepG2 cells [23]. Proteomic analysis revealed differential expression in cell cycle proteins related to G2/M transition (Table 1A). CDK1, 14-3-3σ protein, and annexin-2 decreased in SNU475^PRDX6−/−^. Annexin 2, which also decreased in HepG2 cells [23], forms a complex with 3-phosphoglycerate kinase (PGK), which acts as a primer recognition protein that initiates DNA replication and contributes to cell proliferation [31,32]. In addition, reactome pathway systems analysis of proteomic data showed down-regulation of “the role of GTSE1 in G2/M progression after G2 checkpoint” (Table 1A). Furthermore, SNU475^PRDX6−/−^ cells showed strikingly decreased expression of proliferating cell nuclear antigen (PCNA), a standard marker for proliferation (Figure 4E), and overexpression of PRDX6 in knockout cells returned the levels of PCNA back to those of wild type cells (Figure 4E), showing a direct relationship between the levels of PRDX6 and this cell cycle progression factor.

Enrichment, among down-regulated proteins, of a number of cytoskeleton-related proteins, i.e., the “actinin-type actin-binding”, “filamin-binding”, “tubulin”, and “annexin” domains (Table 2) involved in modulation of cell shape and migration were observed, as well as the formation of stress fibers, which was revealed by phalloidin labeling and a significant increase in cell size (Figure 4F).

**Figure 5:**
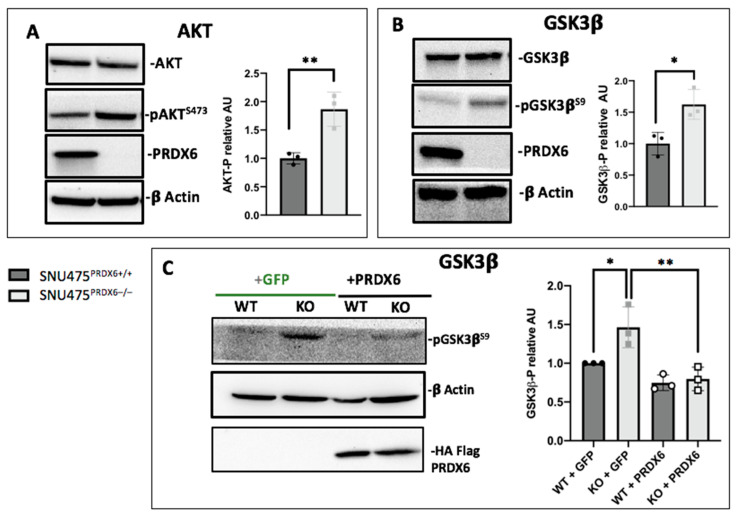
Cell signaling in SNU475^PRDX6−/−^ cells. (**A**) Phosphorylation of AKT determined by Western blot normalized according to AKT total levels and relative to WT (n = 3). (**B**) Phosphorylation of GSK3β determined by Western blot normalized according to GSK3β levels and relative to WT (n = 3); (**C**) SNU475^PRDX6−/−^ cells transfected with *pcDNA3.1 + prdx6* or *pcDNA3.1 + GFP* normalized according to actin and relative to WT (n = 3). Multiple *t*-test, * *p*-value ≤ 0.05; ** *p*-value ≤ 0.005.

Finally, the AKT/GSK3β pathway was analyzed using Western blot. We detected increased phosphorylated AKT in SNU475^PRDX6−/−^ cells (Figure 5A). AKT phosphorylates and inactivates GSK3β at Ser9, and we observed an increased proportion of pGSK3β^S9^ in SNU475^PRDX6−/−^ cells, although the total levels of GSK3β did not change (Figure 5B). The levels of pGSK3β^S9^ reverted to those of wild-type cells when SNU475^PRDX6−/−^ cells were transfected with a plasmid overexpressing PRDX6 (Figure 5C), confirming that lack of PRDX6 is responsible for GSK3β inactivation. The increase in p-GSK3β^S9^ correlates with the activation of MYC, as detected by systems analysis of proteomic data (Table 1C), since GSK3β phosphorylates c-MYC at Thr-58, labeling it for proteolysis [33]. Furthermore, active GSK3β can also phosphorylate cell cycle proteins, promoting cell cycle progression and cell proliferation [34].

These results are supported by the increase in cells at the G2/M phase in knockout cells observed by flow cytometry (Figure 4D).

### 3.6. Peroxiredoxin 6 Deficiency Induced a More Epithelial Phenotype

**Figure 6:**
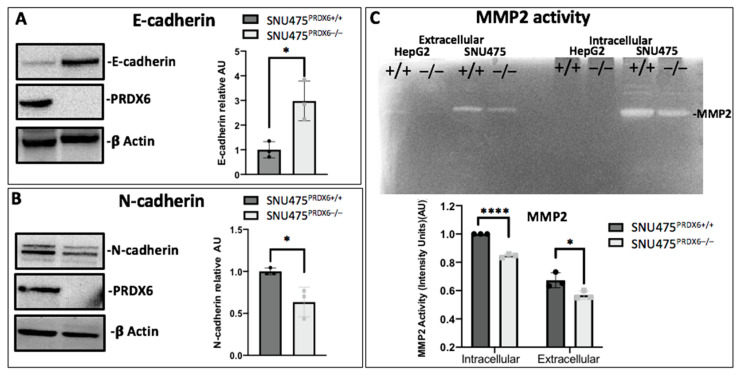
Epithelial–mesenchymal transition of PRDX6-deficient cell lines. (**A**) E-cadherin levels determined by Western blot in HepG2^PRDX6+/+^ and HepG2^PRDX6−/−^ cells, relative to WT and normalized by β-actin levels (n = 3). (**B**) N-cadherin levels in SNU475^PRDX6+/+^ and SNU475^PRDX6−/−^ cells by Western blot relative to WT and normalized to β-actin (n = 3). (**C**) Enzymatic activity of MMP2 (MW = 64kDa) on a gel containing 0.08% gelatin; each lane represents 20 μg of protein from medium (extracellular) and cell extract (intracellular) of HepG2 and SNU475 cells with or without PRDX6 (+/+ or −/−, respectively) (n = 3). (AU = Arbitrary units). (Multiple *t*-test, * *p*-value ≤ 0.05; **** *p*-value ≤ 0.0001).

To study the effect of PRDX6 ablation on EMT, we looked for epithelial and mesenchymal markers. The epithelial marker E-cadherin was increased in HepG2-KO cells (Figure 6A) but was not detected in SNU475-WT cells or SNU475-KO cells. Reciprocally, N-cadherin, a mesenchymal marker, decreased in SNU475-KO cells (Figure 6B) but was not detected in HepG2-WT or HepG2-KO cells. These changes reflect the different phenotypical characteristics of both cell types and demonstrate the functioning of the “cadherin switch” toward more epithelial in HepG2 and less mesenchymal in SNU475 when PRDX6 is eliminated.

Analysis of proteomic data revealed programs of “remodeling of epithelial adherens junctions” (Table 1B) and catenin-∂ (CTNND1) up-regulation in SNU475^PRDX6−/−^ cells (not present in WT) (Supplementary Table S1B), while other cytoskeletal proteins were down-regulated (Table 2), suggesting that PRDX6 promotes the process of dedifferentiation and malignancy of cells through EMT reprogramming. Proteomic data revealed activation of the hepatocyte nuclear factors 4A and 4G (HNF4A and HNF4G) (Table 1C), which have been related to cell differentiation in the liver [35]; therefore, this increase is consistent with the more epithelial phenotype observed in knockout cells.

Cytoskeleton rearrangement of hepatocarcinoma cells, lower MMP2 secretion, decreased migration, and invasion induced by annexin 2 down-regulation have been reported [36]. The SNU475 cell line showed higher metalloproteinase 2 (MMP2) activity than HepG2 cells in cell samples and in cell culture supernatants (Figure 6C), as expected given the mesenchymal phenotype of SNU475 compared to HepG2; more interestingly, when PRDX6 was deleted, MMP2 activity decreased in both SNU475 and HepG2 cell lines (Figure 6C). These data demonstrate that PRDX6 triggers the “cadherin switch” and point to PRDX6 as an important factor in MMP production, both extracellularly and intracellularly, which strongly contributes to cell invasiveness.

**Figure 7:**
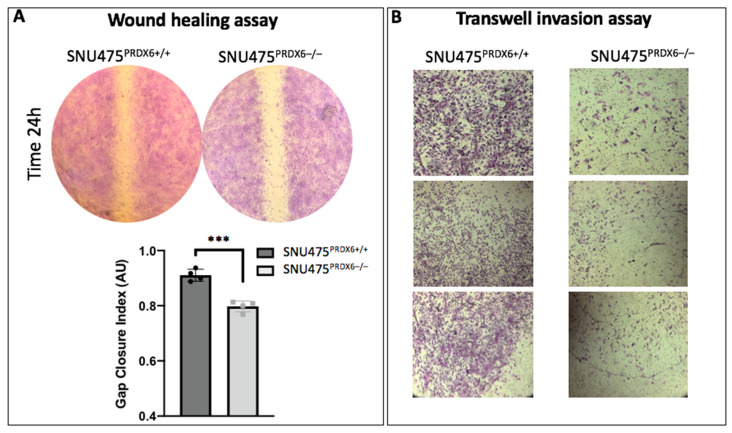
Migration and invasiveness of SNU475^PRDX6−/−^ and SNU475^PRDX6+/+^. (**A**) Representative wound closure assay with SNU475^PRDX6+/+^ and SNU475^PRDX6−/−^ cells visualized at 24 h (**upper**) and quantitation (**lower**) measured by calculating the gap closure index (GCI) (n = 4). (**B**) Representative replicate Matrigel^®^ transwell cell invasion assays using the same number of cells at time 0 and the endpoint at 24 h, showing fixed and stained translocated, invasive cells (upper transwell, non-invasive cells removed). Bright- field images under 10× magnification were taken for SNU475^PRDX6+/+^ and SNU475^PRDX6−/−^ cells. Multiple *t*-test, *** *p*-value ≤ 0.001.

The migration and invasion capacities of the cells were assayed in vitro. Migration assays showed that wound closure after 24 h was lower in SNU475^PRDX6−/−^ cells (Figure 7A). The invasion assay demonstrated a lower number of cells translocated to the bottom chamber under PRDX6 deficiency (Figure 7B), which is consistent with the lower capacity to degrade the matrix due to down-regulation of MMP2. These results were compatible with the inactivation of GSK3β by phosphorylation at Ser 9 (Figure 5B), since GSK3β inhibition was shown to attenuate the invasion of several types of cancer cells by decreasing the secretion and activation of MMP2 [37,38].

Thus, these findings suggest that PRDX6 acts as a regulator of cell differentiation, driving cells toward more pro-metastatic phenotypes through changes in the cytoskeleton, cell shape, motility, and MMP production and activity.

## 4. Discussion

We constructed, for the first time, a PRDX6 knockout cell line in mesenchymal SNU475 hepatocarcinoma cells that, together with a previously constructed PRDX6 knockout in the more epithelioid HepG2 cells, have proven to be useful models that enabled the exploration of different aspects of the influence of PRDX6 that are critical to regulation of the epithelial–mesenchymal transition, which has a role in cancer progression and tumor metastasis. For the interpretation of the data in this study, we assumed the absence of potential off-target effects of the CRISPR-Cas9 technique, based on the clonal nature of the cell line, the consistent results with three different cell lines, and the reversibility of the observed effects upon reintroduction of PRDX6. We showed that knocking out the *prdx6* gene resulted in mitochondrial dysfunction, cell cycle arrest, and diminished proliferation in both hepatocarcinoma cell lines, and decreased the migratory and invasive capacities of SNU475 cells while increasing the epithelial nature of HepG2 cells.

In the present study, we provide evidence that elimination of PRDX6 in the mesenchymal SNU475 cell line results in increased levels of ROS and lipid peroxidation, a likely consequence of loss of the peroxidase activity of PRDX6 and diminished cellular antioxidant defenses through reduced levels of the transcription factor NRF2. Reduction of lipid hydroperoxides by PRDX6 is important in suppressing the pathological effects derived from peroxidation, as well as regulating cellular processes mediated by lipoperoxidation products [9]. Moreover, the product of lipid hydroperoxide reduction by PRDX6, a hydroxy-fatty acid, could play another role in cell signaling [39].

Despite the general idea that tumor cells have defects in mitochondrial oxidative phosphorylation, mitochondrial function appears to be essential for tumorigenesis [40,41]. The increase in peroxidized lipids could be responsible for the activation of ATF4 in SNU475^PRDX6−/−^ cells. ATF4 is a factor that is turned on upon mitochondrial stress, which couples the activation of the integrated stress response with a decrease in mitochondrial function [42]. ATF4 activation promotes the transcription of several cytoprotective genes, which produces metabolic reprogramming toward the synthesis of metabolites such as serine, as in our case, and a proteostatic response that decreases the levels of mitochondrial ribosomal proteins [42]. Thus, oxidative stress caused by PRDX6 deficiency leading to ATF4 activation could be responsible, at least in part, for the mitochondrial dysfunction observed in SNU475^PRDX6−/−^ cells, although GSK3β inhibition plays a fundamental role in mitochondrial dysfunction and cell senescence [43,44] and may also be involved, likely by suppressing the opening of permeability transition pores (mPTPs) [45].

Our proteomic results, further supported by extracellular flux analysis, reflect changes in metabolic fluxes, indicating that glucose does not function as glycolytic or respiratory fuel, since its carbon atoms would not produce lactate or acetyl-CoA, respectively. Carbon atoms could be incorporated into the Krebs cycle via 2-oxoglutarate and acetyl-CoA, which could be provided by fatty acid oxidation, although the ATP yield would be limited by mitochondrial underperformance (Figure 2). This reprogramming of glucose metabolism in SNU475 hepatocarcinoma mesenchymal cells is different from that in HepG2 epithelial cells [23]. However, our observations with both tumor cell lines are consistent in that the lack of PRDX6 caused deficiencies in respiration and glycolysis, which seriously compromised the ability of these cells to divide, migrate, and invade.

Loss of PLA2 activity should be responsible for part of the cell response to the absence of PRDX6. A decrease in the levels of LPC species would explain the suppression of its regulatory action [30] on several genes involved in lipid metabolism in SNU475-KO cells, as revealed by IPA analysis. In addition, LPC is converted by a secreted phospholipase (autotaxin, ATX) to lysophosphatidic acid (LPA), which binds to specific receptors on the cell membrane and mediates cell migration, invasion, proliferation, and survival [46]. Activation of this signaling pathway should be compromised when the production of LPC and LPA is limited, as is the case in SNU475^PRDX6−/−^ cells.

The elimination of PRDX6 from SNU475 cells resulted in the activation of the PI3K/AKT pathway, a canonical prosurvival signature. However, proliferation and growth decreased. On the other hand, inhibition of GSK3β is known to set SNAI1 free to switch on the EMT program, but the opposite effect takes place in SNU475 cells devoid of PRDX6, since cell growth decreases and critical bottom-line functional indicators of EMT are present. Hence, the execution of the AKT prosurvival pathway and the SNAI1-induced EMT program are aborted in SNU475 cells when PRDX6 is absent. These apparently contradictory results on PI3K/AKT and GSK3β are not new. Several lines of evidence have been reported that indicate that over-activation of AKT could have counterproductive effects, inducing growth arrest and cellular senescence, although the mechanisms are poorly understood [47,48]. Regarding GSK3β, its role in cancer is contradictory: The enzyme functions as a tumor promoter or suppressor based on the context, cell type, and phosphorylation status [34]. Its inhibition has been involved in suppression of cell proliferation [49,50] and is the likely cause of CDK1 and PCNA down-regulation and cell cycle arrest in SNU475^PRDX6−/−^ cells [51].

Changes observed in the cytoskeleton, both at cellular and proteomic levels, and decreased secretion and activity of MMP2 should also be responsible for the observed reduced migration and invasiveness potential of SNU475-KO cells. The decrease in annexin-2, N-cadherin, and cholesterol biosynthesis would affect the reorganization and localization of N-cadherin at cholesterol-rich microdomains [52], which would also contribute to diminished migration and invasiveness capacity of cells [32]. Moreover, these changes may also be triggered by GSK3β inhibition, since some of its substrates are proteins related to actin cytoskeleton dynamics, microtubule-associated proteins, and adhesion of cells to the extracellular matrix [53].

Therefore, we suggest that PRDX6 is a GSK3β-mediated regulator of cell differentiation that drives cells toward more malignant phenotypes because of changes in the cytoskeleton, cell shape, motility, and MMP production. Elimination of PRDX6 somehow elicits a response at the level of AKT, which is activated by upstream kinase(s) and maintains its hyperactivation in this cell line, where the presence of the counteracting phosphatase PTEN is negligible, thereby catalyzing phosphorylation of GSK3β at Ser9 and, therefore, its inhibition. The direct elicitor of AKT activation with PRDX6 ablation could relate to increased lipid peroxides or even proper changes in lipid composition and membrane rearrangement upon elimination of the PLA2 activity of PRDX6. For all that, both the peroxidase and PLA2 activities of PRDX6 seem to play a role.

Targeting PRDX6 activity or expression may emerge as a novel therapeutic strategy against hepatocellular carcinoma disease progression.

## 5. Conclusions

Although there is some evidence linking PRDX6 to tumor progression, how it manages to stop tumor growth and metastasis has not yet been described. In this work, we demonstrated that knocking out PRDX6 in SNU475 hepatocellular carcinoma cells prevents cell proliferation, migration, and invasiveness. This is achieved through PCNA down-regulation and AKT-dependent inactivation of GSK3β. Therefore, deletion of PRDX6 could potentially be incorporated as part of a combination therapy approach against hepatocellular carcinoma to impede tumor progression and metastasis.

## Figures and Tables

**Table 1:** Differential proteomics analysis of SNU475^PRDX6−/−^ vs. SNU475^PRDX6+/+^.

**(A) Category**	**Expression Trend**	**Term**	**Count**	**%**	***p*-Value**
**Biological** **Process**	Up	Protein Biosynthesis	12	9.0	1.1 × 10^−7^
	Down	Lipid Metabolism	20	14.3	3.1 × 10^−6^
**Cellular component**	Up	Cytoplasm	83	62.4	3.1 × 10^−19^
		Nucleus	52	39.1	3.2 × 10^−3^
	Down	Cytoplasm	93	66.4	7.6 × 10^−25^
		Cytoskeleton	23	16.4	1.1 × 10^−4^
**Reactome** **Pathway**	Up	tRNA-Aminoacylation	8	6.0	1.7 × 10^−9^
	Down	The Role of GTSE1 in G2/M Progression After G2 checkpoint	11	7.9	3.5 × 10^−9^
**KEGG** **Pathway**	Up	Aminoacyl-tRNA Biosynthesis	8	6.0	7.8 × 10^−6^
		Nucleo-cytoplasmic Transport	9	6.8	2.5 × 10^−5^
	Down	Metabolic Pathways	42	30.0	2.2 × 10^−7^
**(B) Transcrip.** **Regulator**	**Activation** **Score**	***p*-Value**	**(C) Molecular and** **Cellular Function**	**Overlap** **(%)**	***p*-Value**
TP53	−0.943	2.16 × 10^−19^	tRNA Charging	23.1	5.23 × 10^−10^
MYC	2.574	1.54 × 10^−11^	BAG2 Signaling Pathway	13.1	3.69 × 10^−9^
HNF4A	1.213	5.53 × 10^−11^	Coronavirus Replication Pathway	17.18	4.42 × 10^−8^
NRF2	−0.944	3.89 × 10^−10^	Remodeling of Epithelial Adherens Junctions	13.2	9.07 × 10^−8^
ATF4	2.451	1.34 × 10^−9^	Protein Ubiquitination Pathway	5.8	1.49 × 10^−7^
HNF4G	2.828	4.33 × 10^−8^			

(**A**) DAVID enrichment analysis of up- and down-regulated proteins according to proteomic data; the number and percentage of proteins in each category and the *p*-value for statistical significance are shown. (**B**) Ingenuity pathways systems analysis (IPA) of top transcription regulators according to their statistical significance (*p*-value); activation *z*-score is also shown; positive values and negative values indicate activation and inhibition, respectively; (**C**) IPA top canonical pathways according to their statistical significance (*p*-value); overlap indicates the percentage of pathway proteins in the data.

**Table 2:** Cytoskeletal proteins differentially expressed in SNU475^PRDX6−/−^ vs. SNU475^PRDX6+/+^.

Protein IDs	Protein Description	Gene Names	Log FC	*p*-Value
P0DPH8	Tubulin alpha-3D chain	TUBA3D	−2	4.18 × 10^−6^
P08133	Annexin A6	ANXA6	−0.509	0.00032
P09525	Annexin A4	ANXA4	−0.402	0.00155
P50995	Annexin A11	ANXA11	−0.832	0.00315
P07355	Annexin A2	ANXA2	−0.375	0.00924
Q8WUP2	Filamin-binding LIM protein 1	FBLIM1	−0.578	0.00014
P21333	Filamin-A	FLNA	−0.283	0.01528
O75369	Filamin-B	FLNB	−0.207	0.03843
P11047	Laminin subunit gamma-1	LAMC1	−1.704	0.00053
G3XAI2	Laminin subunit beta-1	LAMB1	−1.668	0.02289
O43707	Alpha-actinin-4	ACTN4	−0.140	0.01652

Proteins closely related to the cytoskeleton involved in cell shape and migratory capacity are shown with their UniProt ID, protein and gene names, their fold change expressed in logarithm scale, and statistical significance quantified by *p*-values.

## Data Availability

The mass spectrometry proteomics data have been deposited in the ProteomeXchange Consortium via the PRIDE partner repository with the dataset identifier PXD039491.

## Supplementary Materials

The following supporting information can be downloaded at https://www.mdpi.com/article/10.3390/antiox12061153/s1, Supplementary Figure S1: Construction of a stable SNU475PRDX6^−/−^ cell line; Supplementary Table S1A,B: Total identified proteins (Supplementary Table S1A) and proteins quantified with significant expression changes (Supplementary Table S1B).
